# Autistic-like behaviors are attenuated by agmatine consumption during pregnancy: Assessment of oxidative stress profile and histopathological changes in the prefrontal cortex and CA1 region of the hippocampus 

**DOI:** 10.22038/IJBMS.2023.74536.16190

**Published:** 2024

**Authors:** Khadijeh Mirzaee Khoram-Abadi, Sara Haratizadeh, Mohsen Basiri, Mahdieh Parvan, Fahimeh Pourjafari, Iraj Aghaei, Sedigheh Amiresmaili, Masoumeh Nozari

**Affiliations:** 1 Student Research Committee, Kerman University of Medical Sciences, Kerman, Iran; 2 Department of Anatomical Sciences, Afzalipour School of Medicine, Kerman University of Medical Sciences, Kerman, Iran; 3 Neuroscience Research Center, Guilan University of Medical Sciences, Rasht, Iran; 4 Department of Physiology, Bam University of Medical Sciences, Kerman, Iran; 5 Neuroscience Research Center, Institute of Neuropharmacology, Department of Physiology and Pharmacology, Afzalipour School of Medicine, Kerman University of Medical Sciences, Kerman, Iran

**Keywords:** Agmatine, Autism, Behavior, Pregnancy, Rat, Valproic acid

## Abstract

**Objective(s):**

Due to the crucial role of polyamines during fetal growth and development, we aimed to determine the effect of prenatal administration of agmatine, an endogenous active metabolite of arginine, and a nutritional supplement, on autistic-like behaviors, oxidative-anti-oxidative profile, and histopathological changes of the prefrontal cortex (PFC) and CA1 area of the hippocampus in valproic acid (VPA) model of autism in male rats.

**Materials and Methods:**

VPA was injected intraperitoneally on embryonic days (ED) 12.5, and the pregnant rats were gavaged with agmatine between E6.5 to E18.5 (13 days), at doses of 0.001, 0.01, and 0.1 mg/kg. The autism-like behaviors and memory of male pups were analyzed via open-field, three-chamber, and novel object recognition tests. Serum oxidative stress and the histological changes in the PFC and CA1 were assessed at the end of the study.

**Results:**

The results suggest that prenatal agmatine reduced autistic-like behaviors by decreasing cell loss in CA1 and PFC. We observed no alterations in superoxide dismutase (SOD) level and total anti-oxidant capacity (TAC) between groups. VPA decreased catalase (CAT) activities, while agmatine decreased malondialdehyde (MDA) activity.

**Conclusion:**

Overall, this investigation suggests that agmatine may be a potential candidate for the early treatment and even prevention of appearance of autism symptoms.

## Introduction

Agmatine is an endogenous active polyamine metabolite derived from the amino acid L-arginine (Arg) by arginine decarboxylase (ADC). Under physiological conditions, it is present in small amounts in the central nervous system (CNS) and is considered a neurotransmitter (1, 2). Agmatinergic neurons are primarily located in particular brain regions, including the hypothalamus nuclei, forebrain, and cerebral cortex, which are implicated in various emotional and cognitive functions. Several previous studies have reported the neuromodulatory, antiapoptotic, neurogenic, anti-oxidant, and anti-inflammatory properties of agmatine (3). In addition, agmatine exerts anticonvulsant and anxiolytic effects and has been suggested to be involved in the etiology and pathophysiology of several neurodegenerative and psychiatric diseases (2, 4).

 There is evidence of the role of agmatine in autism spectrum disease (ASD) development as the fastest-growing neurodevelopmental disorder; previous studies have reported that the plasma level of agmatine in patients with ASD is lower than that in the control group. Agmatine is an endogenous N-methyl-D-aspartate (NMDA) antagonist (5, 6), and based on the excitatory/inhibitory imbalance hypothesis of the disease, the acute inhibition of NMDAR action by agmatine normalizes behaviors in adult animals prenatally exposed to VPA (3). In addition, agmatine is a polyamine that plays a critical role during fetal growth and development. Exposure of a growing fetus to a suboptimal uterine condition may respond by developing adaptations that increase vulnerability to a new set of early-onset diseases such as ASD (7).

This study explores the effects of agmatine during the fetal period on the behaviors of pups exposed to maternal valproic acid. Agmatine was administered during gestation, and autistic-like behaviors were assessed in adolescent male rats. The association between VPA consumption during pregnancy and an increased rate of ASD in the progeny is well established, although the biological mechanisms involved and therapeutic strategies remain unknown. VPA impairs the closure of the neural tube and is known as a teratogenic agent. Closure of the neural tube and first neurogenesis occur up to embryonic day (ED)12.5 in the rat, and this time is the most vulnerable time point to induce autism-like behaviors in prenatally VPA-exposed rats. Neural tube defects (NTD) appear as crooked tail phenotypes in rats. 

It is also postulated that VPA may affect the inhibitory switch in γ-Aminobutyric acid (GABA) signaling, and administration of agmatine as an NMDA receptor antagonist during the fetal period (the time of the balance between excitatory/inhibitory systems) improves autistic-like behavior induced by VPA (3, 8, 9). Several studies have revealed that Arg administration improves embryonic survival, growth, and fetal development, whereas no studies have focused on ASD (10, 11). We preferred to use agmatine, decarboxylated Arg, instead of Arg because an advantage of agmatine is its low toxicity, and dietary agmatine sulfate is absorbed in the gastrointestinal tract and is quickly distributed throughout the body, including the CNS. Agmatine is present in maternal and fetal plasma at higher concentrations than other polyamines. It crosses the blood-brain barrier and has neuroprotective, anti-anxiety, anti-depressive, and anti-oxidative effects. Furthermore, increased agmatine secretion following CNS injury suggests that it involves neuro-repair and regenerative processes (1, 7, 12, 13).

## Materials and Methods

In this study, 45 Wistar rats (30 females and 15 males), weighing about 200–220 g, were provided by the Experimental Animal Center of Kerman University of Medical Sciences (Kerman, Iran). All rats were group-housed in a temperature-controlled animal house (22 ± 2 °C, 25 ± 5% relative humidity, and 12:12 hr light/dark cycle), and had free access to water and standard rodent chow. Wistar rats were mated overnight, and the morning when a vaginal plug with spermatozoa was detected was labeled as the gestational day 0.5 (GD 0.5). If a rat did not have a successful pregnancy, it was replaced with another. Finally, 30 pregnant rats were randomly separated into two groups: One group (n=15) was subjected to a subcutaneous injection of 600 mg/kg body weight valproic acid sodium salt (Raha Pharmaceutical Company, Isfahan, Iran) on the embryonic day12.5 (ED12.5), and another group (n=15) served as a non-VPA, which were injected saline (1 ml/kg) (14). From E6.5 to E18.5 (13 days), some of the pregnant rats (n=8) in each group were gavaged with agmatine [(purchased from Sigma-Aldrich; A7127), dissolved in distilled water, 0.0 01– 0.1 mg/kg in a constant volume of 10 ml/kg body weight)].   Some pregnant rats (n=7) were gavaged with distilled water (10 ml/ kg) (15–17). 

The day after delivery, the body weight and crown-rump length of all pups (male and female) were evaluated. The litters were weaned on postnatal day (PND) 21. Only male pups were used in this experiment because previous research has shown that VPA male rats exhibit autistic-like behavior, such as a reduction in sociability, more than female animals. 

2-3 male pups from each mother were included in the study (total n= 56, n=7/group). Behavioral tests were started from PND 40. The time interval between behavioral tests was 24 hr. 

After behavioral assessments, the rats were anesthetized by ketamine/xylazine solution at a dose of 100 mg/kg ketamine and 10 mg/kg xylazine, intraperitoneally. Blood sampling from the heart was then performed to examine serum malondialdehyde (MDA), superoxide dismutase (SOD), catalase (CAT), and total anti-oxidant capacity (TAC). Subsequently, four animals from each group were randomly chosen for hematoxylin/eosin (H&E) staining. The animals were decapitated, and the brains were removed intact.

**Figure 1:**
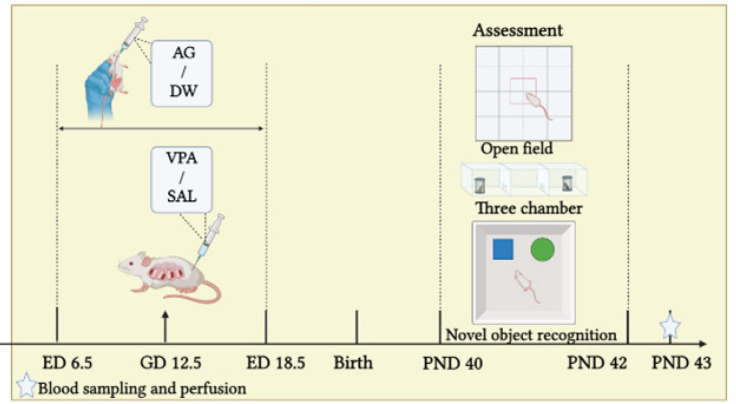
The diagram illustrating the arrangement of the experimental procedure

VPA: Valproic acid; SAL: Saline; DW: Distilled water; AG: Agmatine; PND: Postnatal Day; ED: Embryonic Day

The schematic representation of the experiment’s structure and the representation of the study’s progression are concisely encapsulated in the form of a visual aid, as illustrated in Figure 1. 

The Animal Experiment Committee at Kerman University of Medical Sciences approved all the animal care protocols and experiments (Ethics code: R.KMU.REC.1399.161). They were conducted consistent with the NIH Guide for the Care and Use of Laboratory Animals.

***Behavioral assessments***

*Open field test*

The repetitive behaviors were evaluated using an open field box. A Plexiglas box (30×100×100) was used to confine the animal. The number of self-grooming (rubbing the body with paws or mouth and rubbing the head with paws) and rearing behaviors were noted for 5 min while the animal moved freely. These behaviors were calculated to evaluate stereotypical and repetitive movements in an autism animal model. Of note, a camera was used to record the animal’s behaviors, and then an experimenter blinded to the animal’s group scored these repetitive behaviors (18).

*Social interaction test (three-chamber test)*

Social interaction test (SIT) has been advanced to assess social behaviors in animals. The test was performed at standard room-lighting conditions. The social approach apparatus used for the SIT was a three-chambered rectangular box consisting of a central chamber and two side chambers. The subject animal explored the SIT box freely during three consecutive steps: adaptation, sociability, and social preference. In the adaptation step, each rat was positioned within the central chamber and free to move throughout the chambers to adapt itself to the test environment. The sociability step was started by placing two small wire cages in the mid-section of the side chambers. A novel same-sex and age rat was sited randomly in one wire cage (the novel rat 1). The time spent in each chamber by the subject animal was recorded for 10 min by two separate observers. In the third step, the social preference phase, a new rat (novel rat 2) was placed in the empty wire cage in another side chamber, and the duration of social behavior (the subject investigated novel rats with sniffing, grooming, etc.) was re-recorded for 10 min. 

The sociability index (SI) and social preference index (SPI) were computed using the bellow formula (19): 

SI = (Time exploring novel rat 1-Time exploring empty cage ) / (Time exploring novel rat 1 + Time exploring empty cage)

SPI = (Time exploring novel rat 2-Time exploring known cage) / (Time exploring novel rat 2 + Time exploring known cage)

Note that the known cage refers to the first novel rat. SI and SPI scores are between −1 to +1; values closer to + 1 illustrate more sociability and social preference, while lower points indicate lower indexes (14).

*Novel object recognition test*

The novel object recognition (NOR) test was carried out in a wooden box (60 × 60 × 40 cm) in three steps: adaptation, training, and retention. Each rat was adapted to the empty test box for 10 min in the first step. During the next step, the training phase, the animals were faced (5 min) with two distinct objects, labeled as A and B, at opposing sides of the box, and the investigation time for each rat was recorded. A and B objects had different shapes, colors, and appearances (white cubes and black cylinders) but had equal height (8 cm) and volume. In this stage, the preference index was measured by the following formula: 

Preference index = Time investigating object B/ Time investigating object A + Time investigating object B

The third phase, the testing session, began after about 45 min of the second phase, and object B (cylinder) was replaced with a red pyramidal object 8 cm high (object C). The exploration time was recorded for the known object (A) and novel object (C) for 3 min, and a recognition index was calculated by dividing the duration of interaction with the novel object (object C) by the total time spent on investigation of both objects (objects A and C).

Note that in all stages, the animals could investigate the box freely. Behaviors such as sniffing, rearing, or touching the object along with looking at the object were considered exploratory behavior. All testing apparatuses were cleaned with 70% ethanol. The task procedures were videotaped for data collection (18). 

***Biochemical analysis***

*Malondialdehyde (MDA) measurement*

MDA was assessed using Yagi’s method. At first, 125 μl serum was added to 1.5 ml 1% phosphoric acid and shaken. Next, 0.5 ml of thiobarbituric acid was incorporated, and the mixture was subjected to 45 min heating at 95 °C. After cooling at room temperature, 1 ml of n-butanol was added and centrifuged for 10 min at 7000 rpm. The samples and standards of MDA were eventually evaluated at 532 nm against the blank of the standard curve, and the results were expressed as nmol MDA/ml serum (20).

*Superoxide dismutase (SOD) activity assessment*

The activity of superoxide dismutase (SOD) was ascertained through the implementation of the Randox method (U.K.; Cat NO.RS504). SOD functions as a catalyst in the conversion of superoxide radicals (O2 -) into hydrogen peroxide (H_2_O_2_) and elemental oxygen (O2). In the Randox assay kit, xanthine oxidase (XOD) generates superoxide ions (O2 -) or converts alternatively xanthine to uric acid and hydrogen peroxide, thus causing NBT to NBT-diformazan conversion. NBT-diformazan absorbs light at the wavelength of 560 nm. The concentration of superoxide ions is diminished due to the decreased speed of NBT-diformazan formation by SOD. Consequently, the rate of reduction of superoxide ions present in an experimental sample, in the presence of NBT- diformazan, was used to measure SOD activity (21).

*Catalase (CAT) activity assessment*

The Sinha-modified method of measuring the CAT activity was used. At first, a solution comprising phosphate buffer (50 mM; pH 7.4), 30 mM H_2_O_2_, and the dichromate/acetic acid solution (consisting of a 5% aqueous solution of potassium dichromate in distilled water and 150 ml of Glacial (98-100%) acetic acid) was prepared. Subsequently, the mixture was heated for 10 min in a boiling water bath. A spectrophotometer at 570 nm was used to evaluate the absorbances of samples (22).

*Total anti-oxidant capacity (TAC) serum levels assay*

The ferric-reducing anti-oxidant power (FRAP) method was used for measuring TAC, as shown by Benzie and Strain in 1996. Serum can reduce the ferric-tripyridyl triazine (Fe III–TPTZ) complex to an intense blue, ferrous (Fe II) form at low pH. The determination of blue intensity is contingent on the anti-oxidant capacity of the sample.

 At first, the obtained amount of serum was combined with FRAP reagent, while distilled water was employed as a blank. Following an incubation period of 5 min at 37 °C, the absorbance of the sample was measured at 593 nm. The FRAP values were expressed in micromolar (µM) units (23).

***Histological studies***

The brain tissue samples were immersed in the fixative solution (10% formalin). Subsequently, formalin-fixed tissues were processed in a tissue-processing apparatus. The paraffin embedding and sectioning were carried out by an embedding machine and a rotary microtome (at 5 μm thickness), respectively. Tissue sections were stained using the H&E technique (24).

Intact and degenerated neurons located in CA1, as well as the prefrontal cortex (PFC), were identified and quantified using a light field microscope (Olympus, CX31, Tokyo, Japan) in conjunction with a microscope-connected camera, which provided 40 x magnification. Neurons characterized as intact exhibit a light cytoplasm and a nucleus that appears normal, while those that are degenerated present with an eosinophilic cytoplasm and nuclei that are either shrunken, pyknotic, or karyorrhectic, as observed through the light microscope (25). Cell count was done on four rats from each group and two slides per rat. The results are presented as the percentage of damaged neurons per slide.

***Statistical analysis***

GraphPad Prism 9 (San Diego, USA) was the software used for data analysis. We assessed normality using the Shapiro-Wilk test and presented data as the mean ± standard error of the mean (SEM). The distribution of all data was normal, and a two-way analysis of variance (ANOVA) followed by Tukey’s *post hoc* test (for multiple comparisons between the groups.) was conducted to test statistical significance. Independent variables (fixed factors) were group (saline vs VPA) and treatment (agmatine or DW). Differences in *P*<0.05 were assumed to be significant.

## Results

The mean body weight and mean height (crown-rump length) of all pups (male and female) were mentioned in Table 1.

The administration of agmatine at a dose of 0.001 increased the weight of the pups in the VPA and non-VPA groups [(Saline + 0.001AG versus Saline+ DW, *P*<0.001), and (VPA + 0.001AG versus VPA + DW, *P*<0.001)]. In both VPA and saline groups, when agmatine was gavaged 0.01 mg/kg, the weight gain was less than when it was administrated in the 0.001 mg/kg [(Saline + 0.01AG versus Saline+ DW, *P*>0.05), and (VPA + 0.01AG versus VPA + DW, *P*<0.01)]. Agmatine at a dose of 0.1 mg/kg did not affect weight gain in VPA-exposure animals (VPA + 0. 1AG versus VPA + DW, *P*>0.05) and decreased it in the non-VPA group (Saline + 0. 1AG versus Saline + DW, *P*<0.05). The administration of agmatine did not affect the crown-rump length.

**Figure 2:**
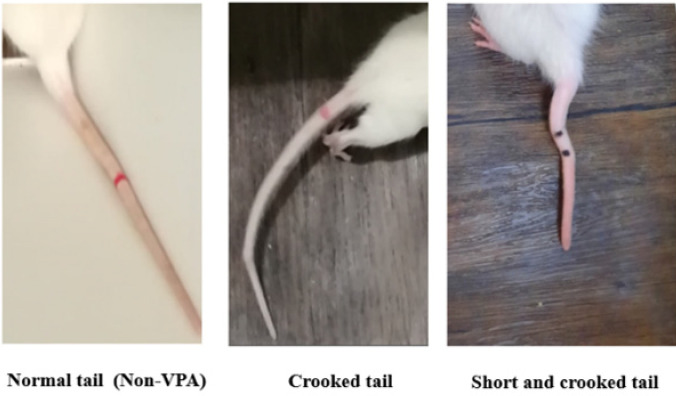
Characterization of tail abnormalities in the VPA-exposed rats. Short and crooked-tail phenotypes were observed

Short and crooked-tail phenotype as a mild form of NTD was observed in all VPA-exposed rats in the current study (Figure 2).

***Effects of agmatine during pregnancy on Spontaneous motor activity***

**Figure 3:**
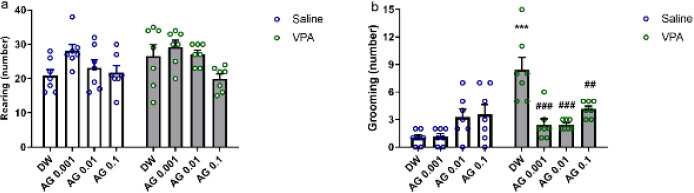
Effect of prenatal agmatine exposure in male pups of VPA- and saline-exposed rats on (a) rearing and (b) grooming

We investigated the impact of chronic agmatine treatment on the rearing and compulsive self-grooming behavior and reported them in Figure 3a-b, respectively.

There was no difference between the rearing in all animals (*P*>0.05, Figure 3a). 

Statistical analysis showed significant effects of VPA injection [F (1,48) = 15.87; *P*<0.001], agmatine [F (3,48) = 5.79; *P*<0.01], and interaction between them [VPA × agmatine: F (3,48) = 11.96; *P*<0.001] on self-grooming behavior. The exposure to VPA resulted in a significant rise in the number of compulsive self-grooming as opposed to the administration of saline (*P*<0.001). Chronic agmatine administration at all doses significantly controlled the grooming numbers in repetitive behaviors in rats treated with VPA (*P*<0.01), while no impact was observed when administered to saline animals (*P*>0.05).

***Effects of agmatine during pregnancy on social behaviors***

The effect of agmatine treatment on social behaviors in the VPA- and saline-exposed rats was evaluated using the SIT.

**Figure 4.**
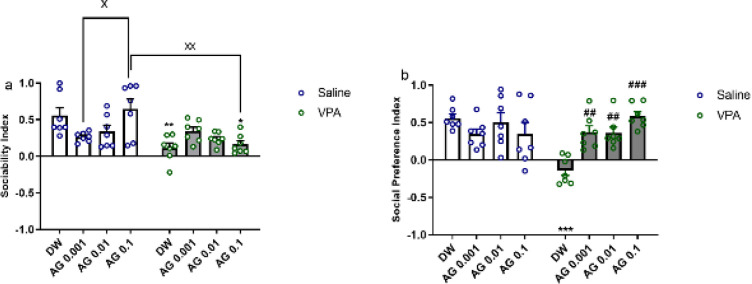
Effect of prenatal agmatine in male pups of VPA- and saline-exposed rats on (a) sociability index (SI) and (b) social preference index (SPI) as evaluated through the social interaction test

During the sociability stage of SIT (Figure 4a), two-way ANOVA analysis of SI data showed significant main effects of VPA [F (1, 48) = 18.36; *P*<0.0001] and VPA × agmatine interaction [F (3, 48) = 6.07; *P*=0.0014]. The VPA-DW animals displayed a noteworthy reduction in the exploring time of the novel rat (SI) compared to Saline-DW rats (*P*<0.01, Figure 4a). At different doses, agmatine did not affect the SI when administered to saline animals (*P*>0.05), and it did not ameliorate the sociability impairments in VPA-treated rats (Figure 4a). 

 In the social preference step of SIT, as depicted in Figure 4b, SPI data indicated significant effects of VPA [F (1, 48) = 4.85; *P*=0.032], agmatine [F (3, 48) = 2.99; *P*=0.039], and the VPA × agmatine interaction [F (3, 48) = 9.31; *P*<0.0001]. 

The saline rats explored the novel rat more than the known rat (SPI= 0.56 ± 0.05, Figure 4b). Conversely, the VPA animals spent less time with the unknown rat (SPI= - 0.13 ± 0.06). The administration of agmatine at the mentioned doses increased the social preference deficit in VPA rats [VPA + AG 0.001 and VPA + AG 0.01 compared with VPA+ DW, (*P*<0.01); VPA + AG 0. 1 compared with VPA+ DW, (*P*<0.001)].

***Effects of agmatine during pregnancy on learning and memory impairment of VPA-exposed rats***

**Figure 5:**
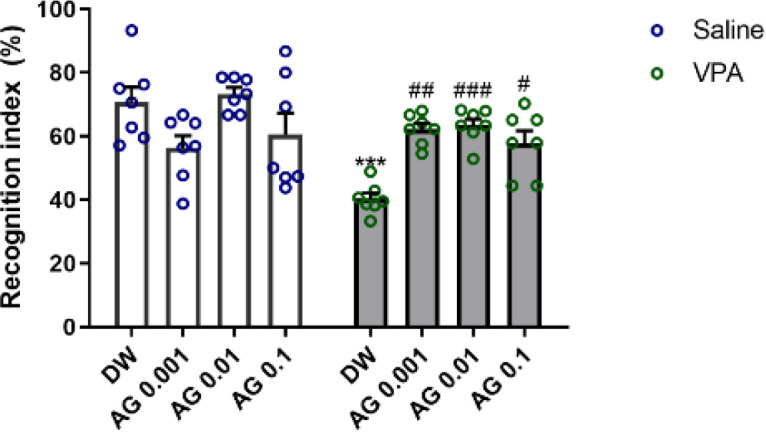
Effect of agmatine during pregnancy on rat male pup novel object recognition impairment induced by maternal VPA treatment

Figure 5 represents the effect of prenatal administration of agmatine on short-term memory as measured by the NOR test.

The NOR preference index during the training phase demonstrated no difference between the groups (data are not shown). 

Two-way ANOVA analysis of preference index during the retention stage showed significant effects of VPA [F (1, 48) = 12.43; *P*<0.0001], agmatine [F (3, 48) = 4.29; *P*<0.009], and VPA × agmatine interaction [F (3, 48) = 8.59; *P*<0.001]. Gestational VPA exposure declined significantly the recognition index compared to saline+ DW (*P*<0.001), revealing a considerable impairment in recognition memory.

No consequential impact of agmatine administration was found when utilized in saline-exposed rats (*P*>0.05). However, agmatine exhibited a significant reversal of the memory dysfunction caused by valproic acid [VPA + AG 0.001 (*P*<0.01), VPA + AG 0.01 (*P*<0.001), and VPA + AG 0. 1 (*P*<0.05) compared with VPA+ DW].

***Effect of treatments on oxidative and anti-oxidative status parameters***

**Figure 6:**
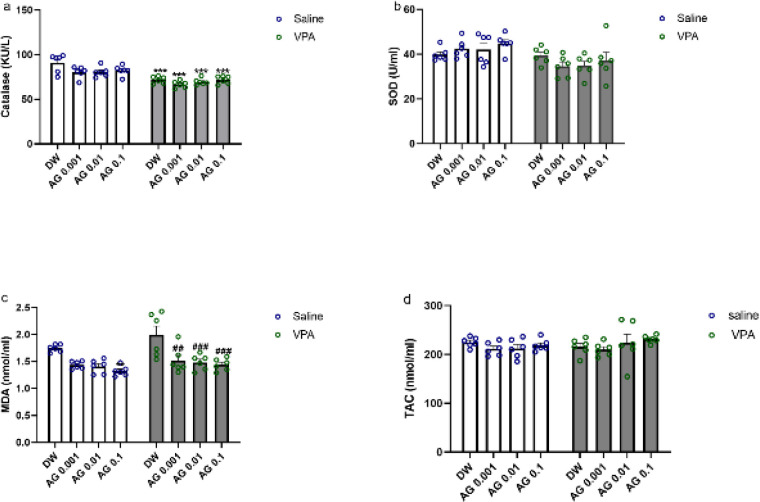
Effect of agmatine on the levels of serum CAT, SOD, MDA, and TAC of male rat pups

Figure 6 depicts the impact of VPA and agmatine on the serum oxidative/anti-oxidative status after gestational VPA or agmatine treatment. VPA+DW animals displayed no notable alterations within the superoxide dismutase (SOD) activity levels, and also, total anti-oxidant capacity (TAC), and malondialdehyde (MDA). 

As regards CAT activity, two-way ANOVA revealed significant effect of VPA [F (1, 40) = 66.27; *P*<0.0001], and subsequent *post hoc* Tukey tests showed that CAT activity was decreased in the animals in the VPA- DW group (*P*<0.0001). Agmatine at all mentioned doses decreased the MDA level (*P*<0.001 compared to the VPA-treated rats that received DW). No other changes were observed.

***Effect of treatments on the percentage of damaged neurons in the CA1 area and frontal cortex***

**Figure 7:**
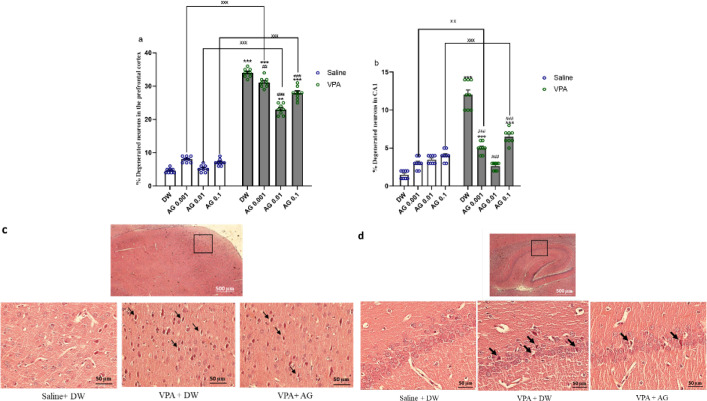
Effect of agmatine during pregnancy on the percentage of damaged neurons in the frontal cortex (a) and CA1 area (b) Higher magnification of the squared area of PFC (c) and CA1 (d) regions exhibits pyknotic neurons, which are shown with arrowheads

The percentage of degenerated neurons in the PFC and CA1 is shown in Figure 7. Two-way ANOVA showed that there were significant differences in the VPA [PFC: F (1, 56) = 4732; *P*<0.0001; CA1: F (1,56) = 233.4; *P*<0.0001], agmatine [PFC: F (3, 56) = 57.50; *P*<0.0001; CA1: F (3, 56) = 47.78; *P*<0.0001], and interaction between them [PFC: F (3, 56) = 55.64; *P*<0.0001; CA1: F (3, 56) = 111.3; *P*<0.0001]. Agmatine at mentioned doses caused a relative reduction in the percentage of degenerated neurons in PFC and CA1. In both areas, there is a significant difference between groups with agmatine treatment and VPA+ DW (*P*<0.01).

**Table 1:** The mean pups’ weight (g) and crown-rump length (mm) of experimental groups

Treatment		Weight (in g; Mean ± SEM)	Crown-rump length (mm; Mean± SEM)	
**Saline + ** **D** **W**		6.23 ± 0.19	4.67 ± 0.08	
**Saline + ** **0.001AG**		8.95 ± 0.23^***^	4.75 ± 0.09	
**Saline + 0.01AG**		6.64 ± 0.08	4.53 ± 0.08	
**Saline + 0.1AG**		5.62 ± 0.10^*^	4.33 ± 0.08	
**VPA+ DW**		5.48 ± 0.10^**^	4.48 ± 0.04	
**VPA+ 0.001AG**		8.68 ±0.09^XXX^	4.56 ± 0.04	
**VPA+ 0.01AG**		6.67 ± 0.04^XX^	4.23 ± 0.06	
**VPA+ 0. 1AG**		5.19 ± 0.09	3.98 ± 0.03	

***P<0.001, **P<0.01, *P<0.1 compared with the control group (Saline+ DW); ^XXX^ *P*<0.001, ^XX^ *P*<0.01 compared with the VPA+ DW. The pups were weighed one day after delivery. The weights are related to all male and female pups

VPA: Valproic acid; DW: Distilled water; AG: Agmatine

Data represent mean ± SEM, n = 7. Statistical analysis was done using two-way ANOVA followed by Tukey’s post hoc test (∗∗∗*P*<0.001, vs Saline-Distilled water (DW); ###*P*<0.001, ##*P*<0.01vs VPA- DW. Distribution of data for individual rats is shown

VPA: Valproic acid; DW: Distilled water; AG: Agmatine

Data represent mean ± SEM (n = 7). Statistical analysis was done using two-way ANOVA followed by Tukey’s post hoc test (∗∗∗*P*<0.001 vs Saline-DW; ###*P*<0.001, ##*P*<0.01 vs VPA-DW; × *P*<0.05, ××*P*<0.01 vs pairwise animals in the saline group). The distribution of data for individual rats is shown

VPA: Valproic acid, DW: Distilled water; AG: Agmatine

The recognition index at the retention phase was impaired in the VPA-exposed group, and agmatine reversed the harmful effects of VPA and increased the novel object exploration. Values are mean ± SEM (n = 7). ***P<0.001 compared to Saline + DW, # *P*<0.05, ## *P*<0.01, ### *P*<0.001 compared to VPA + DW. The distribution of data for individual rats is shown

VPA: Valproic acid, DW: Distilled water; AG: Agmatine

Data are presented as mean ± SEM (n= 6). ∗∗∗*P*<0.001 vs Saline-DW; ###*P*<0.01, ##*P*<0.01 vs VPA-DW. The distribution of data for individual rats is shown

CAT: Catalase; SOD: Superoxide dismutase; MDA: Malondialdehyde; TAC: Total anti-oxidant capacity; VPA: Valproic acid; DW: Distilled water; AG: Agmatine

Treatment with agmatine (0.01mg/kg) only has been demonstrated. ∗∗∗*P*<0.001, **P<0.01 vs Saline-DW; ###*P*<0.01, ##*P*<0.01 vs VPA-DW, ×××*P*<0.001, ××*P*<0.01 vs pairwise animals in the saline group. Hematoxylin-eosin staining: Original magnification 40× and 400×, Scale bar = 50 μm and 500 μm.

The distribution of data for individual rats is shown.

VPA: Valproic acid; DW: Distilled water; AG: Agmatine

## Discussion

The present study investigated the effects of prenatal consumption of agmatine, an endogenous NMDA antagonist, on birth body weight and birth crown-rump length, and also future behaviors of male rats in stereotypic movements, social behaviors, and learning and memory in an autistic model induced by fetus exposure to VPA. Due to the decisive role of oxidative stress on the pathophysiology of ASD, serum oxidative stress status also was assessed. The hippocampus and prefrontal cortex’s histopathological changes were studied because they are extensively implicated in autism. The result showed that in the valproic acid-treated group on day 12.5 of gestational day, a significant reduction in birth body weight without a change in CRL was observed. This weight reduction may be because of VPA’s developmental toxic effects, as Shakya *et al*. reported (26). Eighty percent of prenatally VPA-exposed rat pups showed short and crooked tail phenotypes, which are the specific forms of the NTD process in rodents. The results are consistent with the previous literature (27). Agmatine (0.001 and 0.01 mg/kg) prevented weight loss. It is well known that polyamines are available in large amounts in fetal blood and tissues and have serious impacts on fetal growth and development (7).

Stereotyped behaviors are seemingly purposeless, innate, repetitive, and rhythmic movements that are considered a core symptom of several psychiatric diseases, such as ASDs. Excessive and repetitive self-grooming behavior is a prevalent stereotyped movement that can be seen in various animal models of autism (28). The current study showed significantly higher grooming scores in rats prenatally exposed to valproic acid. The increased grooming behavior of VPA-exposed rats was attenuated by agmatine in all doses tested. According to the excitatory-inhibitory imbalance hypothesis of ASD, we previously reported that early suppression of NMDA receptors by MK-801 during the postnatal period can decrease autistic-like behaviors in the VPA rats (14). Now we evaluate the effect of agmatine as a nutritional supplement and an endogenous NMDA receptor antagonist during pregnancy because it is safe for both mothers and fetuses and can accelerate the inhibitory switch in GABA responses in the fetus’s immature brain (7).Our results also confirm the link between NMDA receptor function and stereotypic behaviors.

Here, we have shown that prenatal VPA impaired social behaviors, and agmatine in this time window could alleviate the social preference deficits. The anxiolytic effects of agmatine have been noted in previous studies, and the social interaction test is a well-validated animal model for assessing anxiolytic compounds (4, 29).

 Prenatal VPA injection impaired the performance of the rats in the NOR test. Poor cognitive outcome in NOR tests is a common behavior in animal models of autism. NOR, as a non-reward and non-spatial memory task, measures animals’ tendency toward new objects versus repeated objects. Previous research revealed links between some neuronal morphological alterations and VPA-induced NOR memory deficits, including reduced cortical thickness, decreased dendritic branching and spine density, and decreases in neurogenesis, cell survival, and proliferation in some brain areas (30, 31). We illustrate that the VPA injection increases degenerated neurons in the CA1 and prefrontal cortex; and agmatine has neuroprotective effects. The common mechanisms for agmatine’s neuroprotective effects include its action as an endogenous NMDA antagonist, α2-adrenoceptor agonist, nitric oxide (NO) inhibitor, voltage-gated calcium channel blocker, anti-apoptotic and anti-inflammatory, as well as, anti-oxidant properties. The present investigation confirms the neuroprotective effects of agmatine (32).

In our study, prenatal VPA increased the serum catalase activity, but agmatine cannot correct it. The concentration of lipid peroxide (MDA) levels was decreased by agmatine treatment. A limitation of our study is that we could not examine serum or brain tissue anti-oxidant profiles during the fetal period, and agmatine effects can be different when the anti-oxidant profiles are measured during the fetal or other ages. Also, the small number of pregnant dams, especially in the VPA group, and the use of pups with the same genetic sources is another challenge of our study that limits the detection of fewer differences between groups.

## Conclusion

We demonstrated that agmatine as a nutritional supplement may help prevent weight loss, increase stereotyped movements, social behavior impairments, and poor performance in the NOR test when consumed during the gestational period in VPA-exposed rats. Agmatine decreased the number of damaged neurons and MDA and may be suggested as an ideal candidate with low side effects for early treatment and prevention of autism. However, the VPA autism model, like other experimental models, possesses distinct restrictions in its applicability to clinical utilization. Subsequently, forthcoming investigations are indispensable in verifying the potential preventive efficacy of agmatine in the context of ASD.

## Authors’ Contributions

K MKA performed data curation, investigation, and methodology; S H helped with writing, review, and editing; M B conceived the study, supervised, and provided the methodology; M P and S A performed investigation; F P and I A provided the methodology; M N conceived the study, supervised, helped with writing, review, editing, project administration, and funding acquisition.

## Conflicts of Interest

None.
